# A qualitative study on clinicians’ perceptions about the implementation of a population risk stratification tool in primary care practice of the Basque Health Service

**DOI:** 10.1186/1471-2296-15-150

**Published:** 2014-09-08

**Authors:** Regina Sauto Arce, Amaia Saenz De Ormijana, Juan F Orueta, Marie-Pierre Gagnon, Roberto Nuño-Solinís

**Affiliations:** O + berri, Basque Institute for Healthcare Innovation, Torre del BEC, Ronda de Azkue, 1, 48902 Barakaldo, Bizkaia, Spain; Osakidetza, Álava-Txagorritxu University Hospital, Jose Atxotegi s/n, 01009 Vitoria, Spain; Osakidetza, Astrabudua Health Center, Mezo 35, 48950 Erandio, Spain; Faculty of Nursing, Université Laval, 2325 rue de l’Université, Québec, Canada

**Keywords:** Stratification, Population health management, Primary care, Implementation, Knowledge translation, Predictive risk models

## Abstract

**Background:**

A prospective Population Risk Stratification (PRS) tool was first introduced in the public Basque Health Service in 2011, at the level of its several Primary Care (PC) practices. This paper aims at exploring the new tool’s implementation process, as experienced by its potential adopters/users, ie. PC clinicians (doctors and nurses). Findings could help guide future PRS implementation strategies.

**Methods:**

Three focus groups exploring clinicians’ opinions and experiences related to the PRS tool and its implementation in their daily practice were conducted. A purposive sample of 12 General Practitioners and 11 PC nurses participated in the groups. Discussions were digitally recorded, transcribed verbatim and analysed by two independent researchers using thematic analysis based on Graham et al.’s Knowledge Translation Theory.

**Results:**

Exploring PC clinicians’ experience with the new PRS tool, allowed us to identify certain elements working as barriers and facilitators in its implementation process. This series of closely interrelated elements, which emerged as relevant in building up the complex implementation process of the new tool, as experienced by the clinicians, can be grouped into four domains: 1) clinicians’ characteristics as potential adopters, 2) clinicians’ perceptions of their practice settings where PRS is to implemented, 3) clinicians’ perceptions of the tool, and 4) the implementation strategy used by the PRS promoter.

**Conclusions:**

Lessons from the implementation process under study point at the need to frame the implementation of a new PRS tool within a wider strategy encouraging PC clinicians to orientate their daily practice towards a population health approach. The PRS tool could also improve the perceived utility by its potential adopters, by bringing it closer to the clinicians’ needs and practice, and allowing it to become context-sensitive. This would require clinicians being involved from the earliest phases of conceptualisation, design and implementation of the new tool, and mounting efforts to improve communication between clinicians and tool promoters.

Graham et al.’s Knowledge Translation Theory proved a suitable framework to explore the implementation process of a new PRS tool in the public Basque Health Service’s PC practice, and hence to identify implementation barriers and facilitators as experienced by the clinicians.

**Electronic supplementary material:**

The online version of this article (doi:10.1186/1471-2296-15-150) contains supplementary material, which is available to authorized users.

## Background

This paper explores Primary Care (PC) clinicians’ perceptions about the implementation of a prospective Population Risk Stratification (PRS) tool in their daily practice in the public Basque Health Service. Prospective PRS strategies and tools are intended to classify individuals according to their predicted future healthcare needs. Information provided by PRS tools serves then to identify subpopulations holding different levels of risk and morbidity burden. This information can then be used as a first step in the development of a population health management approach [1], through the design and implementation of proactive interventions tailored to the needs of each risk group [2]. In the Basque Health Service, a tool for prospective PRS was first applied to targeting interventions in 2011, in the context of a wider regional strategy to improve chronic care [3, 4].

Population covered by the public Basque Health Service (some 2.2 million inhabitants) was stratified using a PRS tool based on the Adjusted Clinical Groups Predictive Model [5]. Following this model, individuals are classified according to the total volume of resources each one of them is predicted to use within the following year. Estimates are based on individuals’ demographic data (age and sex), previous 12 months-clinical data (diagnoses and prescriptions), and history of service use and costs. Results are represented in the form of a predictive risk score, which is calculated as the quotient resulting from the division of an individual's predicted health care costs (in euro) by the predicted average costs for all patients covered by the public Basque Health Service. The prospective stratification tool developed in the Basque Country also allows an easy identification of each individual’s main health problems and offers an estimation of the risk of hospital admission [6]. The owner of the PRS is the Basque Department of Health, which collects and analyses the data and provides the results to clinicians on an annual basis. In the first stages of implementation, O + berri, the Basque Institute for Healthcare Innovation, was responsible for the design and testing of the PRS. It is expected than in the near future clinicians will be able to perform their own analysis and that the update of the database will be done on a quarterly basis. Patients cannot opt out of the database, and all the information is routinely collected by the public administration.

Information generated by the PRS tool is used to identify target populations who could benefit from specific services and interventions. In particular, at the time the focus groups for the present study (December 2011) were run, information had been used to identify candidate patients for secondary prevention interventions to be carried out in all PC practices, as well as for some pilot programmes on case and disease management developed in a few PC practices. In particular, the use that had reached all PC practices was a request by managers to prioritise secondary prevention activities on a targeted population formed by patients diagnosed with hypercholesterolaemia, high blood pressure, or type 2 diabetes, not suffering severe comorbidities (based on criteria as detailed in Table 1) [6]. Indicators on preventive activities on the selected target group were included in the assessment framework used by the general management to evaluate performance by PC practices.

**Table 1:** **Target group of chronic patients with low comorbidity for prioritised secondary preventive activities**

Inclusion criteria	Exclusion criteria
*Presence of one of the following three diagnoses:*	*Presence of one of the following two diagnoses:*
Type 2 diabetes	Heart failure
High blood pressure	Ischaemic heart disease
Hypercholesterolaemia	
*Predicted healthcare needs:*	
Age 0–64 years: predictive risk score <2.7	
Age 65 years or more: predictive risk score <1.5	

General management, as promoter of the new PRS tool, organised informative session with PC unit’s managers (on average a PC unit counts with 13 General Practitioners (GPs)) on this first system-wide use of PRS results in PC. These sessions aimed at raising awareness about the stratification tool and its first application (selected target population, performance indicators…). As for the way results were incorporated into clinical information systems, on the one hand, an icon was added onto the electronic health record of each individual in the target population. On the other hand, GPs were regularly sent out a list of target-patients in their panels.

In this context, the present study aims at exploring the implementation process of the new prospective PRS tool in daily practice of PC in the public Basque Health Service. Despite the growing use of risk stratification tools based on predictive models in various healthcare systems [2], we have not been able to find much literature on the processes of implementation of such tools in daily practice. The few studies that were found are: a report on the use of a stratification tool in Wales [7], a qualitative study carried out in Germany [8], and a survey conducted among doctors in Catalonia (Spain) [9]. Work from Wales presents some Primary Care professionals’ experiences on testing a specific stratification tool, the paper from Germany explores PC physicians’ experiences with case finding through the use of predictive modelling, and the survey in Catalonia explores doctors’ (both practising and managers) opinion about the potential uses of a risk stratification system and its implementation difficulties. Two other papers put up recommendations concerning the implementation of stratification tools based on researchers’ experiences with the use of predictive models [10, 11].

The objective of this paper is to identify the elements that, acting as barriers and/or facilitators, emerge as relevant in the implementation process of a new prospective PRS tool in daily practice of PC in the public Basque Health Service. For this purpose, we explored the perceptions and experience of its potential users/adopters, ie. GPs and PC nurses.

## Methods

The present study draws on qualitative methods to explore clinicians’ perceptions and experiences with the stratification tool implemented in their daily contexts and practices. Qualitative methods are based on an ontological understanding of human realities as complex and plural, which stays in line with basic assumptions of this project: the complexity and plurality of clinicians’ contexts and practices, and thus, perceptions and opinions regarding the PRS tool being implemented.

Researchers considered the dialogical method to be the most appropriate for achieving the study objectives. We chose this method because, considering that the tool was still at an early phase of integration in practice, we were highly interested in understanding the contexts and forms in which the clinicians conceived and gave meaning to it. The specific technique used within this dialogical method was the focus group. In contrast to individual interviews, we believed that focused meetings between peers would better respond to the research objectives by providing a comprehensive view of the different positions (social and professional contexts) in relation to the stratification tool and its implementation in practice.

Participants were selected using a theoretical sampling method, guided by the knowledge translation model of Graham et al. [12, 13] and the findings of previous research in the field of implementation [14]. We identified certain variability criteria, including sex, professional discipline (medicine/nursing), years of experience, profile of individuals as potential adopters of innovations (according to the assessment by managers of their health area) [15, 16], workload (GPs were categorised into equal thirds (low/intermediate/high) by the number of people in each GP’s panel), and the practice setting (rural/urban).

We used Rogers’ Diffusion of Innovation Theory [16] to describe the profiles of participants as potential adopters of innovations. According to Rogers, innovations are adopted following a S curve that plots the rate of adoption by individuals over time. Rogers classifies adopters into five categories. “Innovators” are the first individuals to adopt an innovation, followed by “early adopters” who are the second fastest adopters, and “early majority” who will adopt the innovation significantly later that the previous groups, but before the average. “Late majority” are individuals who will adopt the innovation after the average member of the society, and “laggards” are the last to adopt the innovation. The research team was provided with information on this characteristic for only 65% of the participants, all of them lying between “innovators” and “early majority” profiles.

Three parallel focus groups of PC clinicians were organised by O + Berri, the Basque Institute for Healthcare Innovation. 7 GPs integrated one group (hence called ‘Doctors’ Group’ (DG)). Another group was formed by 7 PC nurses (hence called ‘Nurses’ Group’ (NG)). A mix of GPs (5) and PC nurses (4) made up the third group (hence called, ‘Mixed Group’ (MG)). This form of distribution aimed at exploring differences and commonalities between GPs and PC nurses’ perceptions. Participating clinicians’ characteristics are summarised in Table 2.

**Table 2:** **Characteristics of participants in focus groups**

	Group		
	Doctors	Nurses	Mixed
**Sex**	3 women/4 men	7 women	5 women/4 men
**Practice setting**	Urban: 6	Urban: 6	Urban: 7
	Rural: 1	Rural: 1	Rural: 2
Years of experience*	>25 years: 3	>25 years: 2	>25 years: 4
	10-25 years: 2	10-25 years: 1	10-25 years: 2
	<10 years: 1		
Workload*	High: 2		High: 3
	Intermediate: 3		Intermediate: 1
	Low: 1	Low: 3	Low: 1
**Total**	7	7	9

*Information on these variables is missing for some participants.

Two members of the research team participated in each group, one as facilitator and one as observer. Sessions lasted for approximately 90 minutes. A common theme exploration guide based on Graham et al.’s model [12] was used by facilitators in the three groups. All three group discussions were digitally recorded and transcribed verbatim. A thematic analysis was conducted by two members of the research team (ASO and RS) using NVivo. Thematic analysis aims to identify patterns in the data, and classify them within a limited number of themes [17]. First, the two researchers established independently a list of emerging themes based on the data, compared their results and created a final list of themes. Then, they codified each of these themes into larger categories based on concepts from Graham et al.’ s model [12] (see Additional files 1, 2 and 3). As regards quotes presented in this paper, they have been translated into English from their original Spanish version.

This study received ethical approval by the Clinical Research Ethics Committee of the Basque Country (“Comité Ético de Investigación Clínica de Euskadi’), as part of a larger project (‘Development of Population Risk Stratification Applications’, financed by Kronikgune Research Centre; Grant no.: Kronik11/035). Participants consented to the study by confirmatory email as well as gave oral consent for participation in the study and recording of the conversations.

## Results

In our exploration of the clinicians’ perceptions and experience with the PRS tool, a series of elements shaping the implementation process, and acting as its barriers and/or facilitators, naturally emerged in the discourse by participants in the focus groups. An initial finding from our approach to assessing the PRS tool’s implementation process, from the clinicians’ perspective, is that their views on the new tool and on the implementation process are closely interlinked and influence each other.

The implementation process appears to build up as a complex net of elements, each one of them adopting different forms for each participant, being all at once necessary to understand the implementation process as clinicians’ experience it. Using Graham et al.’s theory as a guide, we present and discuss these implementation barriers and facilitators grouped into the four following categories: 1) elements related to the clinician characteristics as a potential tool adopter, 2) elements related to the clinician’s perception of the practice setting, 3) elements related to the perceived characteristics of the PRS tool and, 4) elements related to the implementation strategy used by the tool’s promoter, as experienced by clinicians.

### Clinicians as potential adopters

The most salient element in relation to potential adopters’ characteristics, and how they influence their perception of the PRS tool, and thus, the tool’s implementation process, is their degree of awareness and alignment with a population health management approach. Participating clinicians showed a wide range of positions in relation to this approach, with those seeming closer to it expressing a more positive perception of the tool. In this sense, a few participants showed a solid public health perspective based on further education and previous experiences with community work. These clinicians thought of a population health management approach as an integral part of their work:

*“"I was working in England for four years and then returned to the Arrigorriaga unit [name of a Basque Health Services’s PC unit] and when I came back, I started identifying needs in my practice setting. The first thing I asked was: Here, what resources do we have, what society, which associations?" (DG6)*

However, even professionals holding more population-health oriented positions found it somehow difficult to apply to their daily practice. Interestingly enough, they were also among those claiming more strongly for organisational changes for such an approach to become part of their everyday reality:

*“…I think that the scarce time I have for individual consultations, maybe I should devote it to patients who are at a level 2 or 3, and not to patients at level 1. But if the patient is hypertensive, even if well controlled, I have to see him/her every 6 months. These are the kind of things that bother me in the daily practice. I think we could better manage our time, but the tools we have somehow force us to follow certain steps…” (DG6)*

Although admitting the need to move closer to this population health management approach, a vast majority of participating clinicians however recognised working according to an individual and reactive approach, which missed acting on those not seeking care:

*“…90% of our effort is for 30% of the population, maybe this is exaggerated, but the patient who does not seek care might be in very bad health. And that's my opinion, we miss delivering prevention to those outside the healthcare circuit” (DG2)*

For this group of participants, the tool seemed of potential use, although they found its implementation somehow far from the possibilities of their everyday practice:

*“…what should I do, what should we do?… I just don´t know, I lack the tools. How can I have an impact?” (NG2)*

*“…I´d love to be able to meet with my diabetic patients and provide them with health education, but I don´t have time, nor the tools, and I´d go as far as to say that I lack the training for it…” (DG4)*

Finally, a minority group of participating clinicians, lacking a population perspective, could only envisage applying the new PRS tool in the context of the individual consultation, finding it of only limited use.

The relevance of further education and experience to be able to put a population approach in practice was also raised (as also shown in last two quotations). Participants recognised their limitations and gaps, especially in relation to facing group and/or community interventions. Clinicians’ resistance to change was also mentioned as an additional barrier to the tool’s implementation, further stressing the need for training:

*“Regarding group intervention, often one departs from his/her own professional resistance. And I speak from personal experience… I struggled, because facing a group of citizens… it’s difficult for the professional” (NG5)*

An element that seemed to act as facilitator of the PRS tool’s implementation was the extent of prior knowledge and information on the tool. Previous direct experience with the tool or with the use of its results, favoured a more positive assessment, and allowed envisioning a broader range of potential applications. Specifically, nurses who had already used lists of target populations resulting from the PRS tool, found it very useful for identifying patients who could benefit from their intervention, but were not yet known/visible to them:

*“I’m finding a lot of people, that is, people I didn´t know myself, who were not coming in for appointments and I didn’t even know how they looked like and, nevertheless,…” (NG4)*

Clinicians’ professional discipline also seemed to influence their perception of the tool, and hence their experience with its implementation process. In this sense, participating nurses, a discipline traditionally further geared to action than doctors, seemed to assess the tool at the very “feet-on-the ground” intervention level, establishing strong links between the tool and the interventions it could be used for. In addition, their interest seemed to focus on community and group interventions:

*“…I would focus more on people who have already been diagnosed, sending them reminders about healthy habits every three months…” (NG2)*

In addition, it was observed that some clinicians showed a special concern about evidence-based practice, questioning about the evidence on the tool’s effectiveness.

### Practice settings where the tool is to be implemented

A large amount of comments by the participating clinicians related to barriers and facilitators in their practice settings. Regarding practice-setting barriers, on the one hand, clinicians referred to an overwhelming individual clinical demand in their daily practice, which prevented them from developing a more proactive role and thus from getting closer to a population health management approach. Patients’ dependency on the healthcare system and resistance to change, within a culture where responsibility for health is seen to rely mainly on healthcare professionals, seemed to further prevent from overcoming this barrier:

*“…Not every problem people may have is to be solved by the [healthcare] system. However, we have created a society where problems must be solved by the [healthcare] system. And there are things that need to be solved by the individual…” (NG1)*

A certain lack of coherence between the evaluation tools used by managers to assess clinicians’ practice performance, and the premises underlying a population health management approach, was also experienced as a barrier to the implementation process. As expressed by participants, assessment tools in place were oriented towards individual activities, and not towards results on population health:

*“…I think that this needs to be clearly set out: [patients] whose condition is well under control don’t really need all the follow-up appointments the system requires us to perform” (DG6)*

Finally a change-saturated practice context seemed to also act as a barrier to the implementation of the new tool PRS in clinicians’ busy practices. The numerous management-driven changes being simultaneously implemented in their practice settings at the time the PRS tool was introduced [3] (although often in line with the participants’ professional views and values), seemed to compete for clinicians’ time and effort:

*“…and so I think that we are in maelstrom of changes at the moment, changes that clearly needed to be implemented, but (in the midst of them) nobody gives us any clear guidance on how to act, or where to start, …” (MG6)*

Participating clinicians also identified elements that, if implemented, would facilitate the new tool’s implementation in their everyday clinical practice. Among these facilitators, clinicians manifested the need for additional organisational changes. On the one hand, as expressed by participants, time limitations could be overcome by reconsidering current assignments and realigning them in coherence with a population health management approach (ie. reducing the time traditionally allocated to certain patients/groups currently receiving very little-impact interventions, in order to increase efforts with patients/groups not yet visible/identified but who might further benefit from clinicians’ attention). The development of new professional roles (already ongoing at the time), in line with further changes in work flows among professionals, and more teamwork, were other organisational changes suggested by participants:

*“I wonder: how can the (Population Risk) Stratification be of use for Primary Care? …First of all, which sort of Primary Care are we talking about? Are we talking about the one currently in place or another sort of it? Is this (PRS) strategy willing to assign everyone, doctors, nurses, new roles? Or not? Shall we be talking about the Primary Care as we know it nowadays, nothing will change, everything will stay just the same as we have seen it since the 80s. Of what use is this to us? So far, of no use, just a new icon to be shown on the screen. Is the system willing to further develop (professional) roles*?” (DG3)

Participating clinicians also pointed at the coordination between healthcare and social care institutions and community organisations (e.g. patients’ associations, community cultural and sport centres, etc.) as a necessary element for promoting the development and adoption of community/population approaches, seen as linked to a more valuable use of the PRS tool.

### The perception of the tool’s characteristics

Participating clinicians identified several elements related to the reliability, desirability and applicability of the stratification tool, which acted as implementation barriers and/or facilitators, depending on the case. So, on the one hand, they questioned the reliability of the tool, mainly as regards the quality of the diagnosis data it builds upon:

*“… it’s based on the ICDs [International Classification of Diseases], but codification is very badly done…” (DG1)*

Its US origin was another issue pointed out when questioning the reliability of the tool, the US healthcare system seen as radically different from the public Basque healthcare system:

*"… Why are we always to be compared to the Americans? What does our healthcare reality have to do with the American one?…What does a person, with free access to healthcare here, have to with an American,…" (NG1)*

Further, the lack of social data in the database the tool relied upon was also mentioned by participating clinicians. They referred to social factors as key determinants of health needs and thus, essential data that the tool needs to contemplate in order to be useful. Particularly nurses strongly stood up at this point:

*“But, which criteria are now being used to identify them (at-risk patients)? Those (patients) not causing any trouble? Those (patients) suffering from one disease? It could be that he/she suffers from 5 diseases but is in great form or getting large support from the private sector… Their (health issues) will not show up in the same way as for other people living in different (social) conditions… I cannot look at them (patients in different social circumstances) the same way, I just don’t look at them the same way” (NG7)*

In addition, clinicians having already been given patients’ lists produced by the tool had been able to identify several errors in the allocation of patients, which eroded their trust.

Participating professionals also talked about several aspects that seemed to limit the desirability of the tool’s implementation in their everyday practice. Firstly, some participants found it difficult to see the advantages of this new tool when compared to other pre-existing tools for identifying target populations. Secondly, in terms of equity in healthcare provision, some clinicians admitted serious worries related to the risks associated to the use of the tool for establishing priorities amongst patients. Thirdly, professionals raised questions regarding the amount and quality of the available evidence concerning stratification-based interventions.

Finally, participating clinicians pointed out several aspects the tool should incorporate in order to make it of easier application to their daily practice, and which could act as implementation facilitators. In this sense, professionals in the groups asked for:

-  more independent management and exploitation of the information by every clinician (especially requested by GPs),
-  further information on patients outside recommended clinical levels and/or those off clinicians’ radar (i.e. those not seeking care),
-  regularly updated information on patients,
-  incorporating social data and information on mental health conditions,
-  a more user-friendly display as for locating patients (at the time it could only be done by patient’s identification code), as well as for alerting clinicians on special issues (patients with values outside recommended clinical levels, with pending interventions…):

*“The only information we have on that stratified patient is an orange icon [in the electronic health record], but, when you open his/her health record, you do not see where you have to intervene, which stratification parameters require intervention, or whether the patients’ values are well-controlled… you have to enter into each medical diagnosis, conduct a data search… it’s not visual, quick or blunt…” (MG5)*

### The promoter’s implementation strategy

The implementation strategy followed by the promoter of a new tool (or innovation), is a critical element in an implementation process [12]. So, the strategy followed by the Basque Health Service’s general management to incorporate the new PRS tool into clinicians’ everyday practice received many comments from the clinicians participating in the focus groups. Three major issues/elements were pointed out (implementation process, information and confidence). All three of them were mutually interrelated and, at the same time, origin and effect of each other. For the purpose of this paper, we shall however present them linearly.

To start with, participating clinicians talked about the process of development and introduction of the new PRS tool in their daily practice, which they considered should have been based on a prior assessment of their practice needs. As they stated it, they were only asked to contribute once the tool was already to be applied. An earlier clinicians’ involvement would have necessarily eased their acceptance of the tool and hence, the tool’s implementation process:

*“… what really strikes me about this meeting here today is how someone who has previously designed something, now comes and asks me about the purposes/utilities of it, the way to use it… whoever designed it must have thought about how it should be used and what for… honestly, this really strikes me…” (NG1)*

When reflecting on how the new tool could be better disseminated across the system, some participants suggested appointing a contact person from the team responsible for the development-implementation of the tool, who would be available for answering clinicians’ questions and concerns.

Participants largely and soundly talked about the information they were provided with. In this sense, participating clinicians clearly showed the relevant role the information seemed to play on the process of accepting and integrating the new tool in their everyday practices. This seemed even more relevant considering that, firstly, target-population interventions emerging from the use of the tool were linked to professional performance assessment. And moreover, results from the PRS tool appeared in the patient’s electronic health record used by clinicians for their daily practice:

*“I thought it was only at my (Primary Care) centre that information had not been received, but I now understand that this (lack of information) was happening all over. We did not have any meeting. This means that, right from the outset, I do not feel comfortable with it, because, as I was just sent a bounced email on the stratified individuals, I don´t know what it´s all about; it´s as if this would not be of my business since I was never invited to get involved” (DG7)*

The facilitating role of the information on the assessment of the PRS tool could also be noticed over the course of the group discussions. In fact, clinicians showing very limited initial knowledge on the tool and its usefulness, as discussions progressed and knowledge was shared, gradually started to envisage a wider range of potential uses.

Finally, participants in the groups extensively elaborated on the level of confidence on the promoter of the tool-implementation (ie. the general managerial level), which also depended on both, the process followed for the tool’s development and introduction in the practice setting, as well as the information on the tool and its objectives and the way it had been provided. Clinicians identified several elements related to building up confidence on tool-promoters:

-  The perceived clarity on the objectives sought by the organisation when introducing changes in clinicians’ contexts of practice. In this sense, linking the implementation of the new tool to professional performance indicators proved not to be an adequate strategy when seeking to increase acceptance. Clinicians would have more easily accepted the tool should they had been provided information in regards to its potential uses when aiming at improving their clinical practice and achieving better population health outcomes. Nurses showed to be especially firm at demanding further information on the goals of the stratification strategy, as well as clearer directions regarding their tasks in relation to the use of the tool.
-  The perceived distance between clinicians and managers, both in relation to managers’ initiatives (not) reaching the frontline level, as well as to the frontline-level work (not) being sufficiently visible and known to managers.
-  The level of evidence on the benefits associated to innovations introduced by managers, with clinicians asking for initiatives to be previously tested and benefits clearly shown.
-  The gradual introduction of innovations, as to have them tested in different contexts and realities within the healthcare system, and to be able to adequately tackle any potential difficulties arising in the implementation process:

*“…before when any new activity was to be introduced…* [examples], *it was first pilot-tested in rural areas, urban areas and other, then difficulties that emerged were monitored and gradually tackled. By the time it was spread out to other centres, problems were still being identified. Nowadays, an innovation is pilot-tested in Bilbao* [urban area] *where not one single issue may come up, but practice in Bilbao is different from practice in “Interior”* [name of a rural health area]*, totally different. It (the innovation) is then introduced in “Interior” health area and it just doesn’t work…” (MG6)*

## Discussion

Graham et al.’s Knowledge Translation Theory [12, 13] has proved to be a suitable framework to explain the implementation process of a new PRS tool in daily PC practice in the Basque Health Service. Our findings also point at the reciprocal relationship between the potential adopters’ perceptions of the implementation process and their perceptions of the new tool (or innovation).

Several characteristics of the potential adopters influence the tool’s implementation process. Clinicians’ values, beliefs and attitudes regarding population health approaches seemed to be among them. So, clinicians showing a stronger population perspective seemed to understand the potential benefits of the tool more easily, and be more favourable to its implementation. Most participating clinicians however saw the need to move closer to a population health management approach. But they recognised that their current practice was geared towards patient-sought individual consultations. In line with these findings, it could be of interest for the success of future implementations of population health management tools, to previously work on bridging the gap between the values underlying the tool and the actual daily clinical practice of their potential adopters. This is likely to require organisational adaptations and further training of staff. Supporting clinical leaders acting proactively and with a population focus could also be of help in signing the way to their colleagues.

Elements related to the practice setting seemed to be of particular relevance in the implementation process. Amongst them, workload, work assignment, roles, and professional performance assessment strategies in place seemed particularly strong in preventing clinicians from adopting more proactive attitudes as well as from undertaking further health promotion activities. It seems then necessary to undertake additional organisational changes and innovations, accompanying the tool’s implementation strategy, that facilitate and provide the necessary tools for clinicians to develop a population health management approach. This finding corresponds to the view of other authors such as Lewis [3], about the need to implement risk prediction tools as part of a wider strategy, as these tools in isolation have no impact on health, rather their effectiveness depends on the interventions performed with the identified patients.

Resistance to change towards a population approach by patients (particularly those for whom services would be reduced) was noted as an additional obstacle. In this sense, it appears that a good communication strategy towards the population and supporting chronic patients develop self-management skills could contribute to adequately overcoming this barrier [18].

The implementation of the stratification tool also depends to a great extent on the way the tool itself is perceived by its potential adopters. Specifically, the clinicians referred to attributes of the tool in relation to its reliability, to the evidence that supports its use, to issues related to the variables included, to its format and ease of use, as well as to the ethical implications of using its results (specifically in relation to equity in the provision of healthcare on the basis of stratification criteria). Other authors have also considered the equity risks linked to the use of predictive models for targeting multimorbid care management interventions [19]. Among the criteria, which seem to generate the most concerns about the reliability of the tool are doubts about the coding of the diagnoses on which the estimates of the model are based, as well as the fact that information of a social nature is not considered. It should, however, be noted that the introduction of this social information was already planned by the developers of the tool, although it was not incorporated in the first version available at the time of the focus groups. Mention was also made (at least among the physicians) to a desire for more independence for each PC team to manage and exploit the information from the stratification tool, as well as a need for the data to be kept up-to-date. These views are in line with those of clinicians in the German study, who also expressed concerns about a time lag between the analysis with the predictive model and the intervention with patients [8]. With respect to complaints about variables, format and accessibility, the tool introduced in Wales seemed to have advanced further, likely due to greater involvement of frontline clinicians in its development [7].

With respect to the aforementioned barriers and facilitators and the elements of the implementation strategy mentioned by the clinicians, it is possible to make a series of recommendations for future implementation strategies for PRS tools. In general terms, these recommendations would aim to bring the tool and its implementation closer to the needs and values of its final users, since, as asserted by Logan et al., the closer to the final user, the more likely that the implementation strategies will be effective [13]. To this end, on the one hand, it seems crucial that clinicians get involved from the early phases of design and implementation of this type of tools, so that they are well suited to their needs and practice settings, before they are scaled up to the entire healthcare system and incorporated into their daily information systems. Moreover, direct experience with the tool seems to improve its assessment by practitioners, as is also shown in other studies on the implementation of risk stratification tools [20]. This would help to foster trust in the tool and in the objectives searched by its promoter. For this, the implementation strategy through pilot site practices used in Wales could be a good model [7], which would also respond to the demands from some clinicians for evidence concerning the positive results of using the stratification tool before its scaling up.

It also seems key to strengthen efforts in communication and provision of information about the tool and its objectives to frontline clinicians (the final users of the tool), especially when the tool requires a change in their usual practice, in this case, from an individual towards a population health management approach. The demand for clarity about the pursued objectives seems to be particularly relevant for nurses. Moreover, this should be a two-way communication, allowing final users to share their impressions and requests in relation to the use of the new tool.

Finally, the implementation strategy should be better aligned with the tool’s goal of promoting proactivity and autonomy by the clinician. Hence, at least in a first phase of implementation of the tool, rather than uses linked to activity indicators common to the entire organisation and established by managers, strategies that allow greater self-management and adaptation to the local context and interests of each PC team would be more likely to be successful (especially among physicians). Such strategies would be supported by a reorientation of the criteria used for the evaluation of PC practice performance towards measuring the impact on population health.

Lastly, among the limitations of this study we should note weaknesses in the method chosen for selecting the participants in the focus groups, especially with respect to their profiles as adopters of innovations [15, 16]. On the one hand, a common tool was not used to classify participants according to their innovation profile and ensure comparability, rather the management of each PC health area characterised their clinicians. On the other hand, it was the managers themselves who decided which clinicians would participate in the focus groups, which could have brought a selection bias towards clinicians who were relatively advanced adopters (from “innovators” to “early majority”). Due to these factors, the results reported here might not represent the realities of clinicians less inclined towards innovation, who also form part of the health system under study. However, we believe that this potential bias is limited because participants in the focus groups showed a wide range of profiles and perceptions towards population management, the stratification tool and its implementation process.

## Conclusions

This study confirms that implementing a PRS tool in PC’s everyday practice is a culturally and organisationally complex process, built upon the intersection of a series of interrelated elements. These elements relate to the characteristics of the potential adopters, their perceptions of their practice settings, their perceptions of the characteristics of the new tool, and their experience with the implementation strategy used by the tool’s promoter.

Several lessons on the implementation of a new PRS tool in clinicians’ daily practice can be drawn from the results of our study. Firstly, in a PC context where an individual approach prevails in clinicians’ everyday practice, the implementation of a population health management tool needs to be framed within a wider strategy promoting a re-orientation towards a population approach. This might require additional organisational changes, such as those identified by the study participating clinicians (clinical practice priorities and assessment tools reoriented, time redistribution, role reassignment, further education and training in population health management and community interventions, increased coordination with social services and community organisations etc.). However, account should also be taken of the risks associated with introducing simultaneous changes in clinicians’ busy daily practices. In this sense, the implementation of a PRS tool within a broader system-level reform strategy would require a substantial effort in planning and communication with frontline clinicians, aimed at avoiding a feeling of change-saturation.

A major issue emerging from the results of our study is the relevance of approaching the new to-be-implemented PRS tool to clinicians’ values, needs and concerns. On this issue, the role of an adequate implementation strategy does not risk being overemphasised. This strategy should not only guarantee smooth communication channels between clinicians and managers, but it should also ensure sensitivity and flexibility to the particular characteristics and circumstances of the diverse practice settings where the tool is to be implemented. This would necessarily mean involving frontline clinicians, as adopters-to-be, right from the very early phases of tool conceptualisation, design and implementation, and allowing them to test and adapt the tool to their own practice settings.

## Electronic supplementary material

Additional file 1: **Doctors’ group node tree [in Spanish].** Node tree resulting from thematic analysis of the Doctors’ group transcribed discussions using NVivo, excel file. (XLSX 9 KB)

Additional file 2: **Mixed group node tree [in Spanish].** Node tree resulting from thematic analysis of the Mixed group transcribed discussions using NVivo, excel file. (XLSX 13 KB)

Additional file 3: **Nurses’ group node tree [in Spanish].** Node tree resulting from thematic analysis of the Nurses’ group transcribed discussions using NVivo, excel file. (XLSX 12 KB)

## Abbreviations

PRS: Population risk stratification
PC: Primary care
GP: General practitioners
DG: Doctors’ group
NG: Nurses’ group
MG: Mixed group.
